# The Immune System in Transfusion-Related Acute Lung Injury Prevention and Therapy: Update and Perspective

**DOI:** 10.3389/fmolb.2021.639976

**Published:** 2021-03-24

**Authors:** Kai Guo, Shuxuan Ma

**Affiliations:** Department of Transfusion Medicine, Beijing Children’s Hospital, Capital Medical University, National Center for Children’s Health, Beijing, China

**Keywords:** transfusion-related acute lung injury, immune system, immune molecule, immunotherapy, prevention

## Abstract

As an initiator of respiratory distress, transfusion-related acute lung injury (TRALI) is regarded as one of the rare complications associated with transfusion medicine. However, to date, the pathogenesis of TRALI is still unclear, and specific therapies are unavailable. Understanding the mechanisms of TRALI may promote the design of preventive and therapeutic strategies. The immune system plays vital roles in reproduction, development and homeostasis. Sterile tissue damage, such as physical trauma, ischemia, or reperfusion injury, induces an inflammatory reaction that results in wound healing and regenerative mechanisms. In other words, in addition to protecting against pathogens, the immune response may be strongly associated with TRALI prevention and treatment through a variety of immunomodulatory strategies to inhibit excessive immune system activation. Immunotherapy based on immune cells or immunological targets may eradicate complications. For example, IL-10 therapy is a promising therapeutic strategy to explore further. This review will focus on ultramodern advances in our understanding of the potential role of the immune system in TRALI prevention and treatment.

## Introduction

Transfusion-related acute lung injury (TRALI) is the onset of respiratory distress and acute lung injury due to blood product transfusion (Semple et al., 2019), and it is a life-threatening complication characterized by the sudden onset of hypoxemic respiratory failure with non-cardiogenic pulmonary edema and bilateral lung infiltration that developed within 6 h of blood transfusion (Toy et al., 2005; Gokhale and Hendrickson, 2019). Although TRALI develops within 6 hrs of blood transfusion, most occurrences take place during transfusion or within the first 1 or 2 h. According to Voelker and Spieth (2019) TRALI is defined as more complex for cases of seeming TRALI, such as transfusion-associated circulatory overload (TACO), and further testing and diagnosis may be required. TRALI pathophysiology has been partially of elucidated. According to the 2004 Canadian Consensus Conference Panel (Kleinman et al., 2004) and TRALI redefinition (Vlaar et al., 2019), TRALI can be identified and diagnosed, and the terms TRALI I [without an acute respiratory distress syndrome (ARDS)] and TRALI II (accompanied by ARDS) have been proposed. However, there were some potential TRALI cases did not meet standard clinical definitions (Fuller et al., 2021).

Currently, TRALI is the leading cause of transfusion-related fatalities (Clifford et al., 2015), and the case fatality rate is 5–10% (Swanson et al., 2006). The incidence of TRALI in surgical transfusion patients is 1.3–1.4% (Clifford et al., 2015), and as many as 15% of patients with severe transfusions develop TRALI (Benson et al., 2010). A report from the US FDA showed that 34% of fatalities were due to TRALI from 2012 to 2016 (fatalities reported to FDA following blood collection and transfusion: annual summary for fiscal year 2016: US Food and Drug Administration), which constitutes one of the leading serious adverse reactions to transfusion. Lieberman et al. (2014) suggested that the incidence of TRALI in children is similar to that in adults. Although the numbers are low, there are no differences in outcomes or presentation between adults and children with TRALI.

Generally, the risk factors for blood transfusion can be divided into antibody-independent and antibody-dependent in the context of TRALI. The former is caused by the transfusion of aged red blood cells or platelets, which contain proinflammatory mediators, bioactive lipids, etc. (Mangalmurti et al., 2009; Vlaar et al., 2010; Peters et al., 2015a). The latter is caused by antibody infusions, which mainly contain human leukocyte antigen (HLA) I and II or human neutrophil antigen (HNA) antibodies (Peters et al., 2015b), are mainly obtained from female donors and cause human neutrophil activation (Popovsky et al., 1983). Antibody-dependent TRALI is the most prevalent type (Popovsky and Moore, 1985; Curtis and McFarland, 2006).

Currently, the two-hit model is used to describe the underlying mechanism of TRALI pathology (Peters et al., 2015b; Lorello and Alam, 2018). The predisposing factors of the patient are the first hit, and antibody or non-antibody factors are the second hit (Silliman et al., 1997; Silliman, 2006). Some clinical studies have shown that an inflammatory first hit almost always occurs before TRALI onset and have identified ARDS risk factors that may also be TRALI risk factors in transfusion recipients, including liver surgery, shock, increased peak airway pressure, chronic alcohol abuse and current smoking in the context of immune system balance and inflammation (Toy et al., 2012). Namely, the clinical condition of the patient induces the release of a large number of cytokines, which cause neutrophil activation, accumulation and adhesion to pulmonary microvascular endothelial cells. The second hit is mainly due to HLA or HNA antibodies from the blood products that directly or indirectly activate the recipient’s immune system. An increasing number of studies have shown that antibody-dependent TRALI is mediated by the activation of neutrophils, which results in the release of oxygen free radicals and proteases, causes endothelial damage, and increases capillary permeability, ultimately causing pulmonary edema, and inducing TRALI (Silliman, 2006).

According to the classic definition, the immune system is comprised of complement molecules, as well as immune cells and their products, including cytokines, chemokines, antibodies, and growth factors. The immune system is considered to be responsible for defending the host from invading pathogenic microorganisms. In fact, the immune system is an integral part of fundamental physiological processes and immune cells function beyond host defense (Sattler, 2017). Researchers in an expanding range of areas are beginning to recognize continuously the implications of the immune system in their respective fields. The immune system plays an essential role in reproduction both before and during pregnancy, and leukocytes are found in male and female reproductive tissues (Oakley et al., 2011; Care et al., 2013). In other words, the effects of the immune system are not limited to host defense but extend to tissue homeostasis, regeneration and the repair of tissues such as the liver (Meijer et al., 2000), kidney (Lin et al., 2010; Zhang et al., 2012), skin (Goren et al., 2009; Mirza et al., 2009), skeletal muscle, heart (Arnold et al., 2007; Nahrendorf et al., 2007; Frantz et al., 2012; Gallego-Colon et al., 2015; Tonkin et al., 2015; Sattler and Rosenthal, 2016), gut (Seno et al., 2009), and brain (Glod et al., 2006; London et al., 2013). Both evolutionary development and functional variety strongly support the idea of the immune system as an all-encompassing system to ensure systemic integrity.

More importantly, the immune response is closely associated with transfusion therapy (Passwater, 2018). Landers et al. (1996) showed the immunomodulatory effects of blood transfusion in 1996. The immune response is an important basis for the occurrence of TRALI (Tung, 2019). Specifically, before and after transfusion, TRALI patients exhibited elevated levels of IL-6 and IL-8, which are the main contributors to the development of TRALI (Roubinian et al., 2015). Fung et al. (2010) also suggested the importance of T cells in reducing the severity of antibody-dependent TRALI in a murine model. Furthermore, it is believed that TRALI is the result of imbalances in the body’s inflammatory response. During infectious diseases, a variety of immunomodulatory molecular mechanisms are activated, including the induction of regulatory T cells (Tregs), regulatory B cells, alternatively activated M2 macrophages or the anti-inflammatory cytokines IL-10 and TGF-β (Taylor et al., 2012; Ziegler et al., 2015; Xu et al., 2016; Loevenich et al., 2017; Soares et al., 2017), which affect all facets of the host immune response to ensure host survival. However, some immune factors, such as B cells, were shown to not play a significant role in the onset of murine antibody-mediated TRALI (Kapur et al., 2017a). Accordingly, in this report, we identified potential immunotherapeutic approaches for TRALI based on immune cells, cytokines and complement molecules by analyzing the literature.

## Regulatory T Cells

Tregs are essential for maintaining immune homeostasis by regulating effector T cell responses, thus facilitating pathogen immune evasion (Velavan and Ojurongbe, 2011) and preventing potential pathogenic host pathogenic effects through a variety of mechanisms (Shevach, 2009; Maruyama et al., 2011; Gao et al., 2012). For example, Tregs express receptor molecules such as CTLA-4, PD-1 and GITR and directly contact target cells to regulate effector cell function, secrete IL-10, TGF-β and other inhibitory cytokines to exert immunosuppressive effects, and allow antigen-presenting cells to enter a non-response state; the expression of enzymes such as CD39 and CD73 indirectly affects the metabolism of target cells, and the secretion of granzymes A and B directly kills target cells and affects immune responses (Shevach, 2009; Josefowicz et al., 2012; Gubser et al., 2016; Rueda et al., 2016). Tregs represent an antigen-specific mechanism to inhibit potentially harmful autoreactive responses.

Although Tregs are defined as T cells with immunosuppressive activity, it has been documented that Treg populations remain diverse (Miyara and Sakaguchi, 2007). Tregs expressing CD25 and Foxp3 are naturally present in the immune system and are considered to be negative regulators of the T cell response. These natural Tregs originate during thymic development (Mold et al., 2008). In addition, other cells, such as IL-10-secreting CD4^+^CD25^–^Foxp3^–^ (Tr1) cells and TGF-β-secreting CD4^+^CD25^–^Foxp3^+^ T cells, have also been shown to exert regulatory effects (Lan et al., 2005). While CD4^+^CD25^–^Foxp3^+^ T cells, which originate from CD4^+^CD25^–^ T cells that develop in the periphery (Wen et al., 2012), CD4^+^CD25^+^Foxp3^+^, CD4^+^CD25^–^Foxp3^+^, and Tr1 Tregs can be activated in different microenvironments. Moreover, CD4^–^CD8^–^ Tregs (double-negative Tregs, DNTregs) express the αβ TCR but do not express CD4, CD8, NK cell surface markers or Foxp3, and CD8^+^ Tregs have no specific surface markers (Lan et al., 2005; Sakaguchi et al., 2010; Murphy and Weaver, 2016). In recent years, a new subpopulation of Tregs (iTR_35_ cells) has been shown to mediate immunosuppression via IL-35 but not IL-10 or TGF-β and is independent of Foxp3 (Collison et al., 2010).

Foxp3-expressing Tregs are known to be indispensable for the maintenance of immunological self-tolerance and immune homeostasis (Sakaguchi et al., 2006). IL-10, TGF-β and Foxp3 are functional and phenotypic markers of natural Tregs (nTregs) and are associated with inducible Tregs (iTregs) (Fontenot et al., 2003). Tregs expressing Foxp3 are able to suppress the activation, proliferation and effector functions (such as cytokine production) of CD4^+^ and CD8^+^ T cells, natural killer (NK) cells, NKT cells, B cells and antigen-presenting cells (APCs) *in vitro* and *in vivo* (Sakaguchi et al., 2008). Tregs can also be isolated and expanded *in vitro*; thus far, treatment with this cell product seems safe and well tolerated (Salas and Panes, 2015).

Notably, some researchers have shown that lymphocytes, especially Tregs, are recruited to the lung in response to lung injury. Tregs may play a key role in protecting against TRALI. Tregs can relieve inflammation by regulating immune responses, thereby alleviating lung injury *in vivo* and *in vitro* (D’Alessio et al., 2009). Venet et al. (2009) confirmed that Tregs reduced neutrophil recruitment and activation by producing IL-10 in lipopolysaccharide (LPS)-mediated acute lung injury. Furthermore, Kapur et al. (2017a) demonstrated for the first time that CD4^+^ CD25^+^ Foxp3^+^ Tregs are critical effectors that protect against antibody-dependent murine TRALI via IL-10. Two years later, He et al. (2019) showed that IL-2c and IL-2 derived from CD4^+^CD25^+^Foxp3^+^ Tregs increased IL-10 and decreased IL-17A, thereby prophylactically preventing antibody-dependent murine TRALI. Subsequently, different subtypes of Tregs, such as Tr1 and iTR_35_ cells, may play important roles in further studies.

## Dendritic Cells

In general, dendritic cells (DCs) are highly specialized antigen-presenting cells that are important in not only initiating immune responses but also in tuning the quality of the immune response or inhibiting the response (Moll, 2003). DCs play a crucial role in this regulatory work, as these cells can regulate T cell-mediated effector responses by generating anti-inflammatory cytokines or inducing Tregs (Maldonado and von Andrian, 2010). Tolerogenic DCs, in particular, can regulate immune responses through various mechanisms (Steinman et al., 2003). Cell fate is predominantly controlled by cytokines in the microenvironment, and to some extent, by the strength of the interaction between the T cell receptor (such as CD45, CTLA-4, and PD-1) and the antigen (Boyton and Altmann, 2002). Tight regulation of effector T cell responses is required to effectively control infections and avoid autoimmune and immunopathological diseases. Aberrant T cell responses, especially those of Th1 and Th17 cells, may play key roles in organ-specific autoimmunity (Dardalhon et al., 2008) and immunopathology (Rutitzky et al., 2008) in the lung. Previously, Kapur R demonstrated that CD11c^+^ DCs protected against antibody-dependent TRALI via IL-10 in mice (Kapur et al., 2017a). The role of DCs may be essential for TRALI immunotherapy.

## Macrophages

Currently, macrophage-targeted therapy has been used in patients (Patel and Janjic, 2015). Alveolar macrophages are central effector cells in the production of proinflammatory mediators. These cells are key in the initiation and resolution of lung inflammation in humans. In the future, the polarization of M1 macrophages may be essential in human TRALI. Lei W et al. (Wang et al., 2020) confirmed that α1-antitrypsin expression improved lung injury by regulating IL-6 production in alveolar macrophages and decreased the M1 macrophage polarization. Modulating macrophage polarization may serve as a potential treatment strategy for human TRALI. By establishing an anti-major histocompatibility complex (MHC) class I monoclonal antibody-induced mouse model of TRALI-like disease, Strait et al. (2011) showed that TRALI induction involves monocytes and macrophages in this murine model.

In addition, osteopontin, a recognized proinflammatory molecule that mediates diverse biological functions (Lund et al., 2009), is involved in various pulmonary disorders, such as fibrosis, inflammation, malignancies, and vascular lung disorders (O’Regan, 2003). Osteopontin is also an important protein involved in regulating the migration of immune cells (Lund et al., 2009). The osteopontin-mediated murine TRALI response is dependent on macrophages, which may be possibly the cellular source of osteopontin (Kapur et al., 2019). In summary, targeting macrophage function may be a critical strategy for TRALI treatment or prevention (Zeeuw van der Laan et al., 2020b).

## Neutrophils

Neutrophils, which have diameters of 12–14 μm, are derived from hematopoietic stem cells. As the first line of cellular defense against invading pathogens, neutrophils can rapidly move across the blood-endothelial cell barrier and exert effector functions during inflammation. Neutrophils are the first responders and are recruited in large numbers to the inflammatory microenvironment by the accumulation of lipid mediators, cytokines, and chemokines, as well as changes in the vascular endothelium (Sadik and Luster, 2012; Mocsai et al., 2015). Neutrophil recruitment to inflamed tissues involves elements of neutrophil rolling and firm adhesion (Filippi, 2019). To capture extracellular pathogen-associated molecules and other stimuli, neutrophils release decondensed chromatin coated with granular proteins, which form neutrophil extracellular traps (NETs) (Branzk et al., 2014; Storisteanu et al., 2017; Watanabe and Petri, 2019). However, the secretion of NETs can also cause tissue damage at the expense of the host and has been shown to impair wound healing or facilitate lung injury in diabetes (Wong et al., 2015) and ventilator-induced lung injury (Yildiz et al., 2015). NETs play an essential role in TRALI. Caudrillier et al. (2012) demonstrated that platelets triggered NET formation in an LPS- or anti-MHC class I antibody-based TRALI mouse model. Thomas et al. (2012) showed that NET biomarkers were present in the serum of TRALI patients, as well as in the lungs of LPS- and anti-MHC class I antibody-induced TRALI mice. Treatment with DNase1 IV, which disrupts the formation of the extracellular chromatin web (Hakkim et al., 2010), was shown to have beneficial effects in these two studies. Although DNases are associated with cystic fibrosis in the lung (Thornby et al., 2014), and additional studies on NETs are necessary in the context of DNase therapy for human TRALI.

In addition, as the major producers of reactive oxygen species (ROS), neutrophils can damage the endothelium in antibody-dependent TRALI mice (Strait et al., 2011; Bayat et al., 2013) and in human pulmonary microvascular endothelial cells *in vitro* (Silliman et al., 2007). Other studies has shown neutrophil-mediated endothelial cell damage in antibody-independent TRALI (Xie et al., 2015) and antibody-dependent TRALI (Nishimura et al., 2006; Silliman et al., 2007; Khoy et al., 2017). Moreover, neutrophils and ROS were shown to be critical in antibody-mediated murine TRALI, as demonstrated by *in vivo* neutrophil depletion and the use of gp91phox-knockout mice, respectively (Kapur et al., 2017a).

Lipids in blood products also damage endothelial cells via neutrophils in an antibody-independent TRALI model, and in human pulmonary microvascular endothelial cells that were activated by LPS and cocultured with neutrophils (Wyman et al., 2002). Neutrophil-dependent endothelial cell damage also occurred in the presence of soluble CD40L (Khan et al., 2006). The Fcγ receptor on neutrophils can recognize the Fc-tail of IgG, which binds to pathogens via the IgG-Fab region (Vidarsson and van de Winkel, 1998; Hogarth and Pietersz, 2012; Kapur et al., 2014). A study by Looney MR (Looney et al., 2006) showed that neutrophils and their Fcγ receptors were essential in a TRALI mouse model. Moreover, researchers found that neutrophil Fcγ receptors were critically involved in TRALI, as adoptive transfer of wild-type neutrophils into TRALI-resistant Fcγ receptor-knockout mice ameliorated acute lung injury upon challenge with anti-MHC class I antibodies (Looney et al., 2006). Neutrophil depletion can protect against anti-HNA-3a antibody-mediated TRALI in mice, although TRALI was not completely prevented in this context (Bayat et al., 2013).

Neutrophils are key effectors in TRALI pathogenesis (Rebetz et al., 2018). Pulmonary neutrophil infiltration has been shown to occur in multiple murine TRALI models (Looney et al., 2006, 2009; Kelher et al., 2009; Fung et al., 2010; Tung et al., 2011; Semple et al., 2012; Bayat et al., 2013, 2015; McKenzie et al., 2014; Kapur et al., 2015, 2017a, 2018) and human TRALI patients (Dry et al., 1999; Silliman et al., 2005; Akagi et al., 2020). Currently, new evidence clearly points to the plasticity of neutrophils and may present a new strategy for the potential treatment of TRALI.

## IL-10

IL-10 is a member of the IL-10 family of cytokines and a non-covalent homodimeric alpha helical cytokine with structural similarities to IFN-γ. The IL-10 receptor (IL-10R) is expressed on the surface of most hematopoietic cells, including T and B cells and macrophages. Genetically, the immunological and physiological functions of IL-10 were found to be non-redundant in engineered mouse models that lack IL-10 or IL-10R. IL-10 inhibits the proliferation of CD4^+^ T cells and the secretion of many kinds of cytokines (Fiorentino et al., 1989).

IL-10 expression is altered in patients with TRALI relative to that in control patients. Kapur et al. (2017b) showed reduced IL-10 levels in patients with TRALI. In all pulmonary transfusion reactions, the combination of clinical variables and cytokine measurements indicated optimal diagnostic performance, and the model comparing TACO and TRALI correctly classified 92% of cases relative to expert panel diagnoses (Roubinian et al., 2015). Low plasma IL-10 levels were associated with TRALI susceptibility in a mouse model, and IL-10-knockout mice were also hypersensitive to TRALI induction (Kapur et al., 2017a). These results suggested that low IL-10 levels were a risk factor for TRALI in humans (Kapur et al., 2017b). However, despite this beneficial effect, these data were in contrast to the findings in patients who suffered from other inflammatory/transfusion-associated pulmonary disorders, such as sepsis-associated acute lung injury (Kapur et al., 2017b) or transfusion-associated circulatory overload (Roubinian et al., 2015), in which IL-10 levels were often elevated. This finding may indicate that TRALI has a distinct mechanism of injury and may be uniquely characterized by impaired IL-10 production. However, two reports demonstrated that IL-10 levels were increased in TRALI patients (Looney et al., 2006, 2014). However, the study showed the changes that may indicate risk factors before blood transfusion (Looney et al., 2014) and may explain this discrepancy.

Regardless, IL-10 infusion may have a therapeutic effect in human TRALI and may completely prevent and protect against the development of antibody-dependent TRALI without any apparent side effects both prophylactically and therapeutically by ameliorating the TRALI reaction in mice (Kapur et al., 2017a). Furthermore, the administration of IL-10 to healthy volunteers was documented as a safe intervention, and only mild to moderate side effects, such as flu-like symptoms, were observed (Moore et al., 2001). We therefore hypothesize that IL-10 administration may be an attractive approach for alleviating TRALI, and clinical studies in humans are highly warranted within 6 h of transfusion for acute lung reactions. However, IL-10 may impair host immune system resistance to infection in clinical settings.

In summary, low plasma IL-10 levels were associated with TRALI susceptibility in a mouse model, and IL-10-knockout mice were also hypersensitive to TRALI induction (Kapur et al., 2017a). IL-10 levels are low in TRALI patients (Kapur et al., 2017b), and very importantly, IL-10 therapy both prophylactically and therapeutically alleviates TRALI in mice (Kapur et al., 2017a). Therefore, IL-10 therapy is a promising therapeutic strategy to explore further.

## IL-8

IL-8 is a CXC chemokine that promotes neutrophil chemotaxis and degranulation downstream of CXC-chemokine receptor (CXCR) 1 and CXCR2, which are G protein–coupled receptors (Holmes et al., 1991; Hammond et al., 1995; Waugh and Wilson, 2008). A study by Roubinian NH demonstrated that IL-6 and IL-8 were elevated in patients with TRALI before and after transfusion relative to those in the control group (Roubinian et al., 2015). In H-2Kd/H-2Dd antibody-based severe combined immunodeficient TRALI models, it was demonstrated that the binding of H-2Kd/H-2Dd antibodies to monocytes increased the levels of macrophage inflammatory protein 2 (MIP-2), causing increased pulmonary neutrophil infiltration and subsequently inducing TRALI (McKenzie et al., 2014).

*In vivo*, peripheral blood monocyte depletion or chemokine blockade completely stopped TRALI induction using an MIP-2 receptor antagonist in severe combined immunodeficiency (SCID) mice (McKenzie et al., 2014). Roubinian et al. (2015) showed that IL-8 is a known risk factor for TRALI, and blocking the IL-8 receptor may also be sufficient to suppress neutrophil chemotaxis and degranulation, thereby counteracting TRALI induction. Moreover, in clinical trials, CXCR2 antagonists were evaluated in other pulmonary disorders, such as cystic fibrosis, asthma and chronic obstructive pulmonary disease (Chapman et al., 2009; O’Byrne et al., 2016), and may have potential efficacy in TRALI as well.

## Complement Cascade

One hundred years ago, the complement system was defined based on its ability to “complement” the antibody-dependent and cell-dependent immune responses against pathogens in blood-based antimicrobial system to compared that of global regulators of immunity and tissue homeostasis. Today, many studies have focused on understanding the biology of complement and its mechanisms of action. Our understanding of innate immunity has substantially changed. Complement plays an essential role in modulating innate and adaptive immunity, supports innate immune responses and the initiation of general inflammatory reactions, contributes to tissue and organ development and promotes tissue repair after injury. In cooperation with other immune and physiological systems, the complement system is key in the recognition and elimination of invading pathogens and mediates the clearance of apoptotic cells, cellular debris and immune complexes to remove self-derived danger signals by integrating innate and adaptive immunity (Ricklin et al., 2010; Arbore et al., 2017).

Distinct mechanisms of the complement cascade are triggered by classic, lectin or alternative signals that converge at the third component (C) and lead to the generation of effectors that make up or supplement the ability of antibodies and phagocytes to clear microbial intruders via the opsonization of C3b, which promotes inflammation via anaphylatoxins C3a and C5a and lyses susceptible pathogens via the C5b-9 membrane attack complex (Ricklin et al., 2010). Complement C5a was shown to play an important role in an anti-MHC class I antibody–based TRALI model in BALB/c mice (Strait et al., 2011). Strait et al. (2011) confirmed that the anti-MHC class I antibody-induced murine TRALI model involved complement activation and C5a production (Strait et al., 2011). However, Looney et al. (2006) described the induction of TRALI 2 h after anti-MHC class I antibody injection in C5a receptor-deficient mice (Looney et al., 2006), and additional validation is needed on the role of C5a as a possible TRALI therapy before the use of C5 inhibitors (Nunn et al., 2005). This further research will shed light on the relevance of the complement system in TRALI, which may open up new therapeutic avenues to explore in combatting TRALI. Cleary SJ demonstrated that complement depletion protected the lungs in endothelial MHC I-deficient mice, and targeting complement cascade activation may be useful for TRALI treatment or prevention (Cleary et al., 2020). Zeeuw van der Laan et al. (2020a) further confirmed that TRALI-inducing murine antibodies have increased abilities to activate the complement system, especially Fc-mediated complement activation, compared to those of antibodies that do not cause TRALI. Complement activation by the endothelium initiates antibody-dependent acute lung injury. Neutrophil responses, including NET release, were intact in endothelial MHC I-deficient mice, whereas complement depletion reduced lung injury (Cleary et al., 2020).

In summary, the complement cascade eradicates invading microorganisms, apoptotic cells, and immune complexes. This evidence should significantly increase our understanding of the role of complement in TRALI and thereby potentially result in promising new treatment strategies. Additional systematic studies are needed to elucidate the role of complement in TRALI (Jongerius et al., 2019).

## Other Factors

McKenzie et al. (2014) demonstrated that intact antibody-induced TRALI was abrogated after the depletion of peripheral blood monocytes. However, TRALI was restored upon purified monocyte restoration. Looney et al. (2009) showed that platelet depletion and aspirin treatment protected mice in an antibody-dependent TRALI model. Cognasse et al. (2020) further showed that although platelet depletion did not prevent the occurrence of TRALI in a mouse model, it did reduce TRALI severity and inhibit TRALI development, and recipient platelets are likely involved in TRALI (Semple and Kapur, 2020). The blockade of sphingomyelinase, extracellular vesicle elimination, or supplementation with sphingosine-1-phosphate during platelet storage may present promising new TRALI prevention strategies (McVey et al., 2021).

We should also note that C-reactive protein (CRP) enhances antibody-mediated TRALI induction in mice (Kapur et al., 2015) and that CRP levels are elevated in human TRALI patients (Kapur et al., 2016). Some studies have indicated that targeting the CD40-CD40L interaction could be an important method to prevent or protect against TRALI (Tariket et al., 2019; Hu et al., 2020). The importance of the gut flora in TRALI induction (Kapur et al., 2018) is continuously being studied and explored.

In addition, cytokines connect and interact with each other through a crosstalk network. Cytokines play important roles in regulating the immune response, differentiating and developing immune cells, and mediating the inflammatory response. The TRALI model shows that host inflammation develops, and the disease is the first blow, causing endothelial cells to secrete cytokines, which helps the lung attract neutrophils by increasing the surface expression of cell adhesion molecules. Lung endothelial cells release cytokines and chemokines to activate and induce neutrophils to accumulate in pulmonary capillaries, stimulating neutrophil-mediated endothelial cell interactions and mediating lung injury. Therefore, other than IL-10 and IL-8, other cytokines may be also potential research targets for TRALI prevention and treatment. For example, Qiao et al. (2020) preliminarily confirmed that IL-35 could prevent murine TRALI by inhibiting the activation of endothelial cells. The study of additional immunological factors in combination with previous research findings could shape the field of TRALI research. For example, IL-6, TNF-α, IL-1β and danger-associated molecular patterns (Land, 2013; Tolle and Standiford, 2013; Liu et al., 2017) are factors that induce ARDS.

## Discussion

**FIGURE 1:**
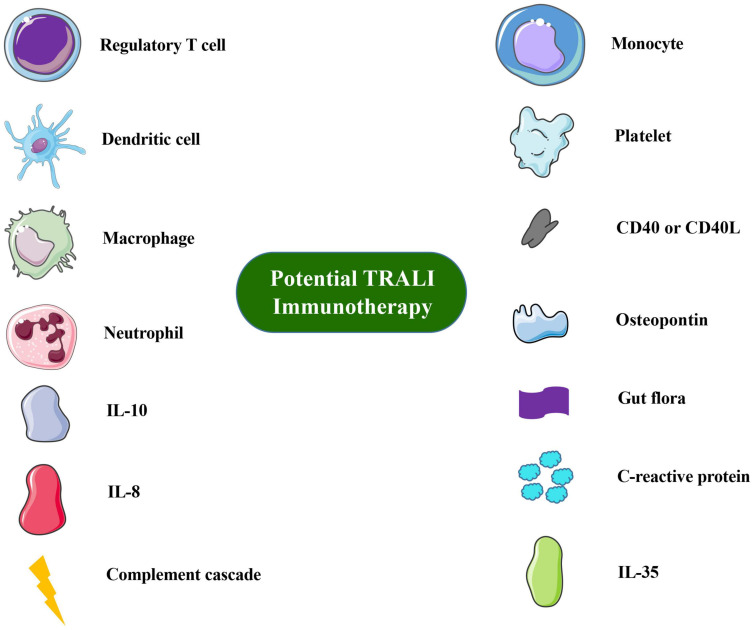
Overview of immune cells or molecules involved in TRALI immunotherapy or prevention. Images of cells and molecules were in part produced or modified using the Smart Servier Medical Art (https://smart.servier.com/), which is licensed under a Creative Commons Attribution 3.0 Unported License (https://screativecommons.org/licenses/by/3.0/).

Overall TRALI remains a significant clinical problem. The development of TRALI animal models is highly important in dissecting the disease pathology because factors other than volume appear to play a significant role in TRALI. Based on previously studies, these immune approaches may significantly contribute to combating these life-threatening complications of blood transfusion. Cell-based therapies, such as those based on Treg or DC administration, seem to be safe and well tolerated. Cytokines or secreted factors have increasingly been utilized to validate risk factors for TRALI, and cytokine concentrations are related to the pathogenesis of TRALI. As Semple et al. (2018, 2019) proposed, the most promising therapeutic strategies to explore are interleukin-10 therapy, down-modulating C-reactive protein levels, targeting reactive oxygen species, or blocking the interleukin-8 receptor. Innate immune molecules, such as complement, are also important. The immune cells or molecules involvement in TRALI are summarized in Figure 1.

These studies focused on the immune system and may provide potential new therapeutic strategies for TRALI. It will be important to expand the investigation of immune-mediated treatments for the clinical diagnosis of and decision making regarding TRALI patients.

## Author Contributions

KG and SM conceptualized the review. Both authors wrote the review, prepared the figure, and edited and approved the final manuscript.

## Conflict of Interest

The authors declare that the research was conducted in the absence of any commercial or financial relationships that could be construed as a potential conflict of interest.
