# Corrigendum: The Immune System in Transfusion-Related Acute Lung Injury Prevention and Therapy: Update and Perspective

**DOI:** 10.3389/fmolb.2021.720653

**Published:** 2021-08-27

**Authors:** Kai Guo, Shuxuan Ma

**Affiliations:** Department of Transfusion Medicine, Beijing Children’s Hospital, Capital Medical University, National Center for Children’s Health, Beijing, China

**Keywords:** transfusion-related acute lung injury, immune system, immune molecule, immunotherapy, prevention

In the original article, there was a mistake in Figure 1 as published. The image representing the C-reactive protein was an inaccurate illustration. Additionally, in the caption, there was an absence of the method used or the source description for the production of the image elements. The corrected Figure 1 and caption appears below. 

**FIGURE 1:**
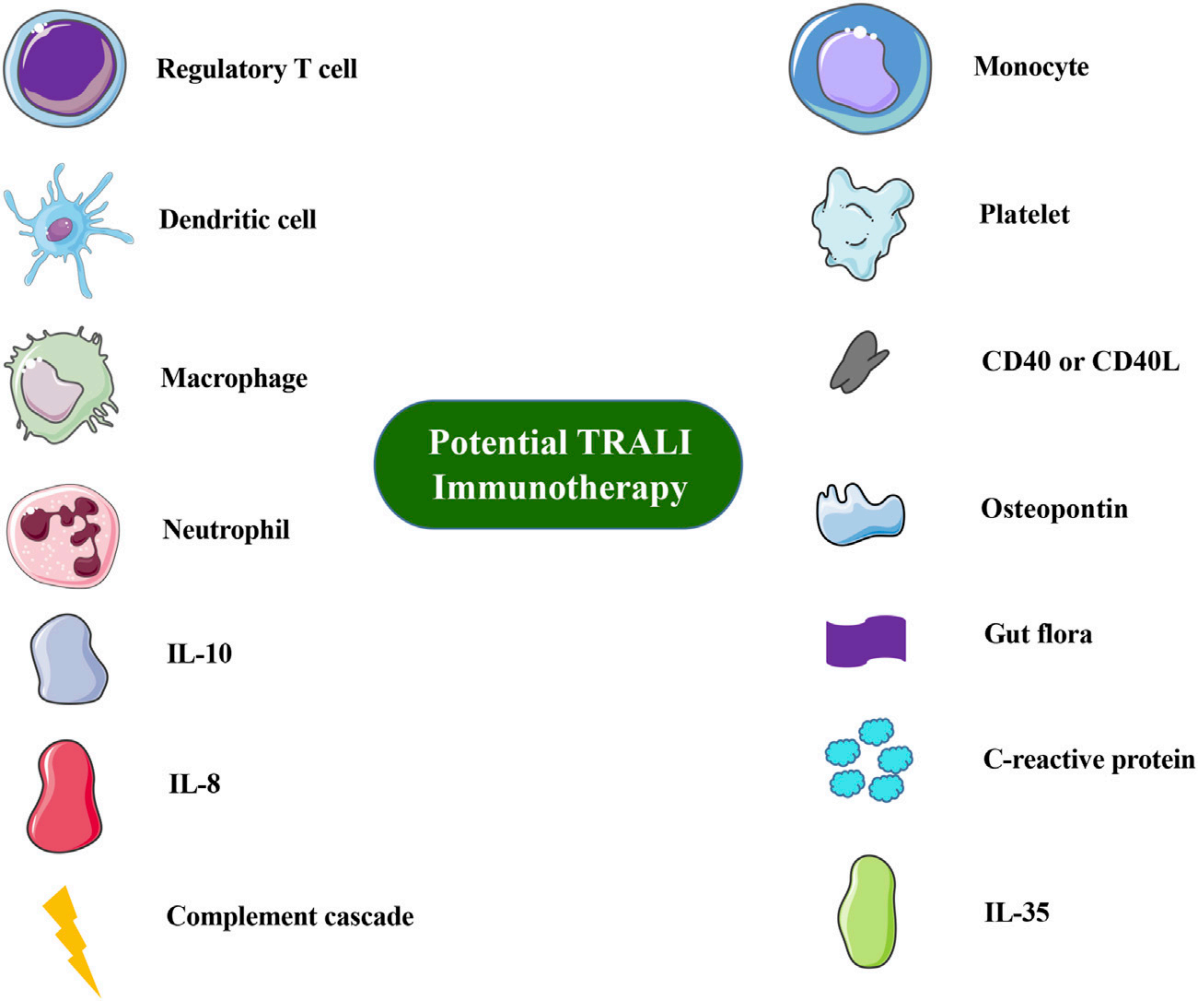
Overview of immune cells or molecules involved in TRALI immunotherapy or prevention. Images of cells and molecules were in part produced or modified using the Smart Servier Medical Art (https://smart.servier.com/), which is licensed under a Creative Commons Attribution 3.0 Unported License (https://creativecommons.org/licenses/by/3.0/).

The authors apologize for this error and state that this does not change the scientific conclusions of the article in any way. The original article has been updated.

